# Supplementary material for: Stacking Model‐Based Classifiers for Dealing With Multiple Sets of Noisy Labels
Source: Biom J. 2025 Mar 12;67(2):e70042. doi: 10.1002/bimj.70042 (PMC11898607; doi:10.1002/bimj.70042)
Supplement: Supplementary file 1 — Supporting Information [file BIMJ-67-e70042-s001.zip › density-based-ensemble-model-main/competitors_real_data/netlab-master/netlab-master/help/mdndist2.htm]

Netlab Reference Manual mdndist2


# mdndist2

## Purpose

Calculates squared distance between centres of Gaussian kernels and data

## Synopsis

```
n2 = mdndist2(mixparams, t)
```

## Description

`n2 = mdndist2(mixparams, t)` takes takes the centres of the Gaussian
contained in
`mixparams` and the target data matrix, `t`, and computes the squared
Euclidean distance between them. If `t` has `m` rows and `n`
columns, then the `centres` field in
the `mixparams` structure should have `m` rows and
`n*mixparams.ncentres` columns: the centres in each row relate to
the corresponding row in `t`.
The result has `m` rows and `mixparams.ncentres` columns.
The `i, j`th entry is the
squared distance from the `i`th row of `x` to the `j`th
centre in the `i`th row of `mixparams.centres`.

## See Also

`mdnfwd`, `mdnprob`

---

**Pages:**
Index


---

Copyright (c) Ian T Nabney (1996-9)

David J Evans (1998)
